# Neurotoxoplasmosis in a Patient with Chronic Lymphocytic Leukemia

**DOI:** 10.5334/jbsr.2475

**Published:** 2021-05-20

**Authors:** Astrid Moraine, Frdric London, Olivier Lebecque

**Affiliations:** 1 Universit catholique de Louvain, CHU UCL Namur, BE; 2 Universit catholique de Louvain, CHU UCL Namur, Department of Radiology, 1 Avenue Dr G Thrasse, 5530, Yvoir, BE

**Keywords:** Computed tomography, vasculopathy, dissection, occlusion, haemorrhage, acute abdomen

## Abstract

**Teaching Point:** Neurotoxoplasmosis should be part of the differential diagnosis for single or multiple cerebral lesions in hematologic patients.

## Introduction

Neurotoxoplasmosis is the most aggressive presentation of toxoplasmosis and is associated with high mortality. It usually occurs in immunocompromised patients, with hematologic patients representing an emerging group at risk. We describe a case of neurotoxoplasmosis in a HIV-negative patient with chronic lymphocytic leukemia (CLL).

## Case Report

**Figure 1:**
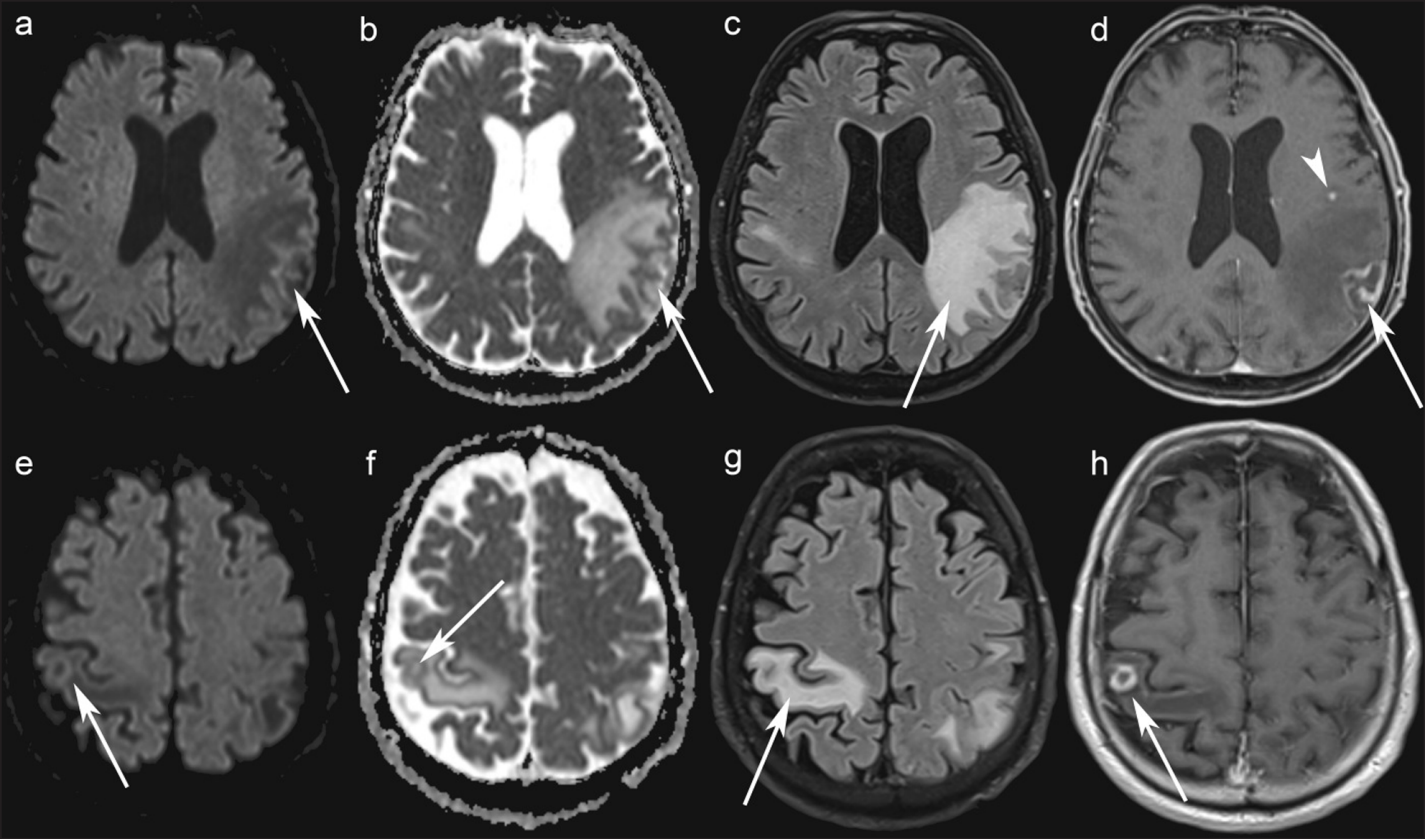
Brain MRI. Axial Diffusion-Weighted Imaging (b = 1500 s/mm) show no increased signal in the left and right parietal lobe lesions (**a, e**) while axial ADC maps show a greater signal than that of the unaffected white matter (**b, f**) (arrows). Axial FLAIR images show edema around the lesions (**c, g**). Axial contrast-enhanced T1-WI shows rim-enhancing lesions in the left and the right parietal lobes (arrows) (**d, h**), and one punctate lesion in the left frontal lobe (arrowhead) (h).

**Figure 2:**
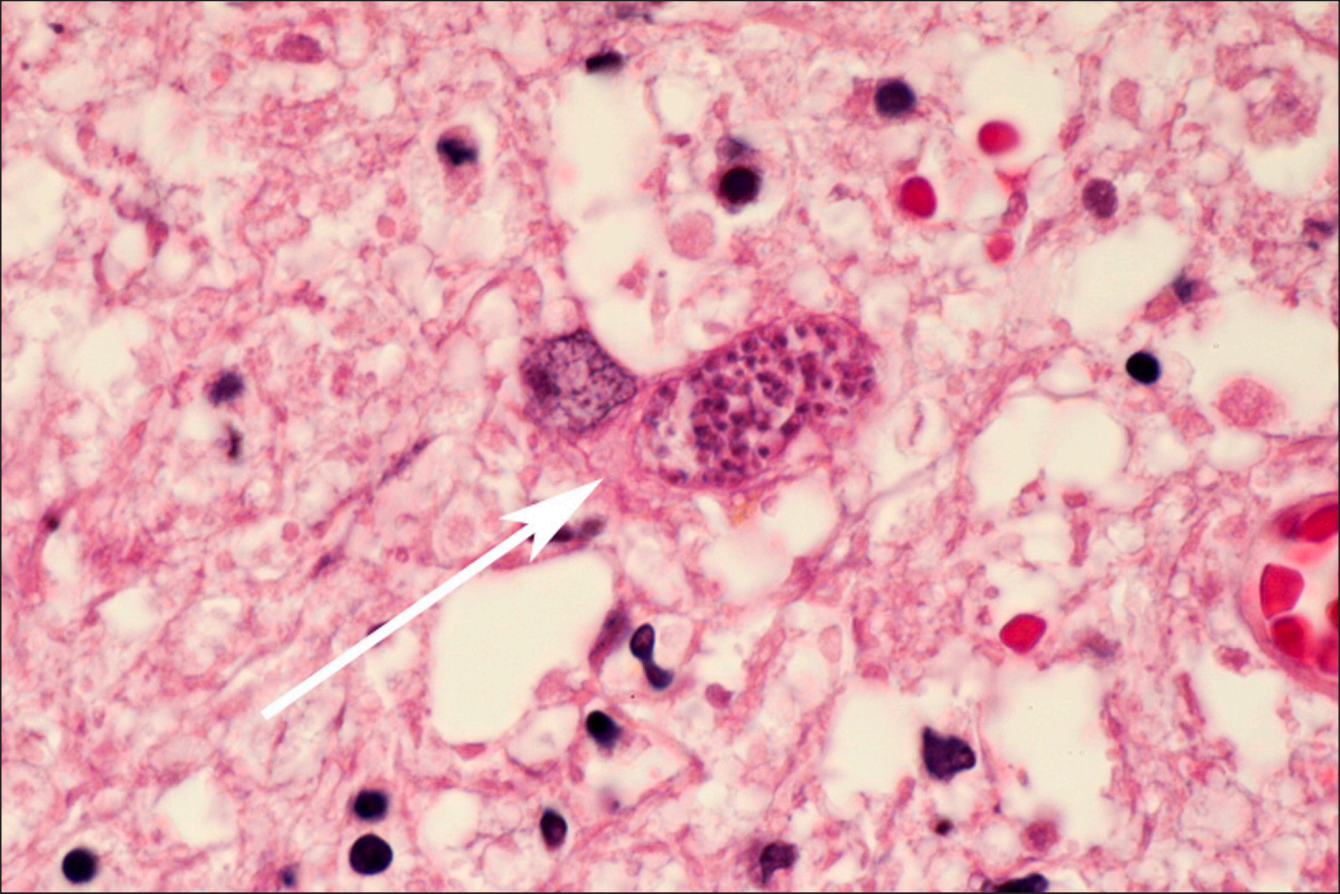
Histological examination (hematoxylin and eosin stain, 100) shows Toxoplasma gondii pseudocyst consisting of multiple protozoa within a cell (arrow).

A 76-year-old man was admitted to the hospital after he developed dysarthria and confusion over the last three weeks. His medical history was remarkable for CLL, diagnosed 12 years prior. Recent history included neutropenia, thrombopenia, hypogammaglobulinemia, and obinutuzumab-chlorambucil chemotherapy (suspected cause of the cytopenia [1]). The patient was afebrile. Neurological examination revealed left-sided hemiparesis and aphasia, but no meningeal signs. Laboratory results revealed a leukocyte count of 1.01 10^3^/l (normal range, 3.79.5 10^3^) and increased C-reactive protein (14.6 mg/L). Toxoplasma gondii IgG was positive (IgM negative), indicating past infection. HIV test was negative. Brain MRI was performed, and gadolinium-enhanced T1-weighted images (WI) revealed three rim-enhancing lesions, the main one measuring 27.5 mm (***Figure 1***). Five focal areas of enhancement were scattered throughout the cortex. There was important vasogenic edema appearing as large areas of hyperintense signal involving the white matter around the main three lesions on both fluid-attenuated inversion recovery (FLAIR) and T2-WI. There was no restricted diffusion within the lesions, making the diagnosis of multiple cerebral pyogenic abscesses unlikely. Metastatic disease was suspected, but whole-body 18-fluorodeoxyglucose positron emission computed tomography did not detect any distant primary malignancy. Cerebrospinal fluid (CSF) examination disclosed mild pleocytosis and elevated protein level, but PCR for Toxoplasma gondii was negative. Surgical brain biopsy was performed, and histopathological examination revealed Toxoplasma pseudocysts consistent with cerebral toxoplasmosis as a result of reactivation of latent infection (***Figure 2***). Intravenous co-trimoxazole was prescribed, and the patients neurological status improved rapidly.

## Discussion

Neurotoxoplasmosis remains a common cerebral opportunistic infection in patients with acquired immune deficiency syndrome. This case emphasizes the need to consider toxoplasmosis in immunocompromised patients with hematological malignancies, representing an emerging group at risk [2,3,4,5]. Early diagnosis is essential to ensure early treatment, but clinical presentation of cerebral toxoplasmosis is unspecific [4]. Immunosuppression and delay of antibodies appearance can be responsible for false-negative serologic results in immunocompromised hematologic patients [2,6]. Brain MRI often demonstrates rim-enhancing cerebral masses on contrast-enhanced T1-WI [7,8]. When present, the eccentric target sign, a ring-shaped zone of peripheral enhancement with an eccentric nodule along the wall on post-contrast T1-WI is considered highly suggestive of toxoplasmosis [9]. Perilesional edema is common [7,10]. A T2-WI/FLAIR target sign with a hypointense core and an hyperintense intermediate region and a peripheral hypointense rim has been described [8]. The core of a rim-enhancing Toxoplasma abscess shows no restriction of water diffusionunlike pyogenic abscessand may resemble a metastasis or a primary brain tumor on diffusion-weighted MRI [7]. Intralesional susceptibility signal foci on susceptibility-weighted imaging, likely representing hemorrhage, have been reported to be present in most patients [11]. Brain biopsy with histological examination can provide a definitive diagnosis [2,12].

## Conclusion

In summary, brain MRI can show rim-enhancing Toxoplasma lesions with no restriction of water diffusionunlike pyogenic abscessmimicking metastases or primary brain tumors. While commonly encountered in HIV-positive patients, neurotoxoplasmosis should also be considered in immunocompromised patients with hematological diseases.
